# Effects of different interchain linkers on biological activity of an anti-prostate cancer single-chain bispecific antibody

**DOI:** 10.1186/s12976-015-0010-5

**Published:** 2015-08-06

**Authors:** Chao-hui Hao, Qian-he Han, Zhong-jie Shan, Jian-ting Hu, Nan Zhang, Xue-pei Zhang

**Affiliations:** Department of Urology, Zhengzhou People’s Hospital, No.33 Huanghe Road, Jinshui District, Zhengzhou, 450003 People’s Republic of China; Department of Urology, The First Affiliated Hospital of Zhengzhou University, No.1 East Jianshe Road, Erqi District, Zhengzhou, 450052 People’s Republic of China

**Keywords:** Interchain linker, Anti-γ-seminoprotein, Anti-CD3, scBsAb, Prostate cancer, Biological activity

## Abstract

**Background:**

A single-chain bispecific antibody (scBsAb; an engineered antibody), has promising clinical applications. Nonetheless, the effect of different interchain linkers on its activity is poorly understood.

**Methods:**

Gene synthesis was used to splice the anti-γ-seminoprotein single-chain antibody (anti-γ-Sm scFv) gene with the anti-CD3 single-chain antibody (anti-CD3 scFv) gene via different interchain peptide linkers. The Phyre2 software was used to predict spatial configuration of different scBsAbs. Eukaryotic expression vectors carrying scBsAbs were constructed by molecular cloning techniques and these plasmids were transfected into HeLa cells with liposomes. scBsAbs were purified by Ni^2+^-NTA agarose and analysed for antigen binding by an enzyme-linked immunosorbent assay (ELISA). Blood pharmacokinetics and inhibition of prostate tumour growth in nude mice were analysed in in vivo experiments.

**Results:**

Bioinformatics analysis and prediction showed that none of the three linkers, Fc, 205C’, and HSA, had a significant effect on protein folding of anti-γ-Sm scFv or anti-CD3 scFv. Nevertheless, the spatial structures of the three linkers were noticeably different. Anti-γ-Sm × anti-CD3 scBsAb with an Fc, 205C’, or HSA linker was successfully constructed, and these antibodies had similar protein expression levels. ELISA showed that all the three scBsAbs bound to Jurkat cells and the LNCaP membrane antigen, although binding of (205C’)scBsAb was weaker than that of the two parental scFvs (*P* < 0.05). In contrast, binding strength of (HSA)scBsAb and (Fc)scBsAb was close to that of the parental scFvs (*P* > 0.05). Pharmacokinetic analysis showed that the half-clearance time of the elimination phase (T_1/2β_) for (HSA)scBsAb was the longest: up to 4.4 h. Compared with γ-Sm ScFv, the three scBsAbs all had a much stronger inhibitory effect on the growth of prostate cancer (*P* < 0.05), but there were no significant differences among the three scBsAbs (*P* > 0.05).

**Conclusions:**

HSA is the optimal linker for the anti-γ-Sm × anti-CD3 scBsAb and may improve antigen-binding affinity of antibodies and prolong physiological retention time. Interchain linkers affect the function of scBsAbs; these effects may have important implications for construction of antibodies.

## Background

Prostate cancer, with such features as a long incubation period and high incidence, ranks the second among male malignant tumours in terms of incidence [1]. The conventional treatments for prostate cancer include surgery, corticosteroids, radiotherapy or chemotherapy. With the deepening of anti-tumor immune mechanism research and the discovery of a variety of tumor-associated surface antigen, the clinical trials for prostate cancer immunotherapy have been widely carried out. Currently, cytokines are the most involved in prostate cancer immunotherapy such as interleukin-2 (IL-2) and granulocyte macrophage colony-stimulating factor (GM-CSF) [2]. Cytokines can be applied as immunoadjuvants, recombinant proteins independently or combines with different tumor-associated antigens (TAA) to prompt the specific anti-tumor immune response. Besides, pre-clinical trials have demonstrated that the vaccine based on prostate specific antigen (PSA) can stimulate humoral and cellular immunity [3]. γ-Seminoprotein(γ-Sm) is the specific antigen secreted by a prostate tumour and is located in prostate cancer cells and their metastases. It is the specific biomarker of prostate cancer and is used for diagnosis and treatment of this disease [4, 5].

Targeting of a tumour-related antigen is the starting point of tumour immunotherapy. Researchers try multiple methods to modify antibody molecules to enhance their function [6, 7]. It is now a hot topic in this field of research to link a single-chain antibody with other effector molecules to construct fusion proteins with anti-tumour properties. Accordingly, a bispecific antibody (BsAb) is one of directions in this field aimed at improvement of tumour immunotherapy via engineering of antibodies [8]. A BsAb contains two kinds of specific antigen binding sites that can build a bridge between tumour cells and immune effector cells and thereby to trigger a cytotoxic reaction and launch targeted killing of the tumour cells [9]. Mashall et al. [10] designed and constructed a fusion protein of anti-ErbB2 scFv and CD28; this fusion protein could be used for targeting of breast cancer cells positive for ErbB2 expression, providing a stimulatory signal for activation of T cells Report of Vaishampayan et al. [11] provided a strong rationale for developing phase II trials to determine whether ATC armed with Her2Bi (aATC) are effective for treating castrate resistant prostate cancer.

A single-chain bispecific antibody (scBsAb) is expressed as a single-chain bispecific molecule because researchers linked the genes of different single-chain antibody fragments through a peptide linker at the genetic level [12]. Due to the covalent bond between different antibody fragments, a scBsAb is rather stable and easy to overexpress in various expression vector systems. Currently, there are several amino acid sequences available as linkers for construction of scBsAbs. Mallender et al. [13] designed the linker CBH124 amino acid residues long. Gruber et al. [14] used 205C’ (25 amino acid residues) to construct an anti-T-cell receptor × anti-fluorescence scBsAb. Such interchain linkers may help each component of a scBsAb to fold correctly and preserve the binding affinity for the corresponding antigens.

Extensive research into the effects of interchain linkers on biological activity of scBsAbs may facilitate identification of an optimal linker and construction of a scBsAb. In the present study, we designed and constructedanti-γ-Sm × anti-CD3 scBsAbs with different interchain linkers. We examined the effects of different linkers on antibody expression, antigen binding, and metabolic characteristics in vivo as well as the inhibitory action on prostate cancer.

## Results

### Analysis of three-dimensional conformation of scBsAbs

**Fig. 1:**
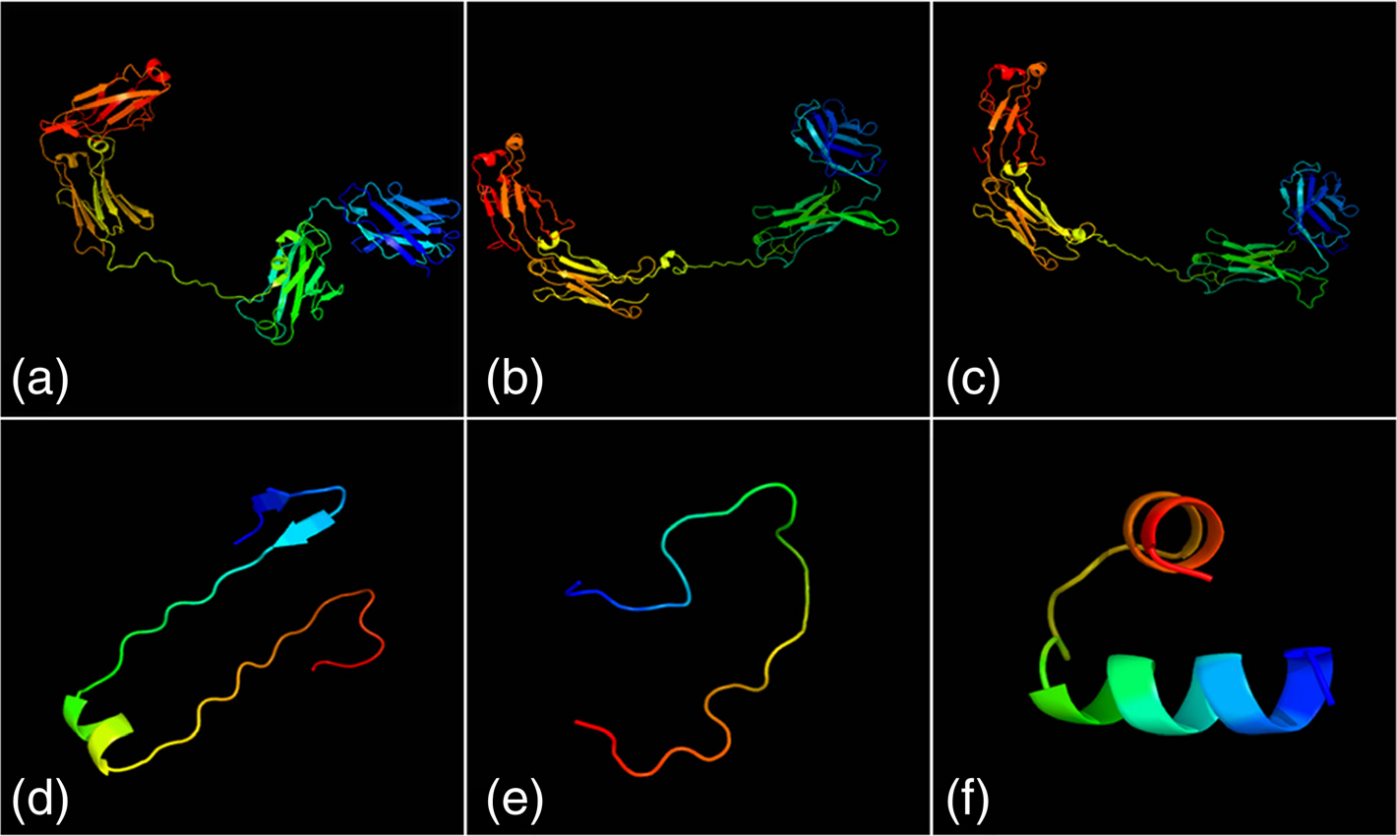
Prediction of tertiary structure model. **a**, **b**, **c**: Tertiary structure respectively for (Fc)scBsAb, (205C’) scBsAb and (HSA) scBsAb; (**d**, **e**, **f**): Tertiary structure respectively for intrachain linkers Fc, 205C’ and HSA

Results of prediction by the Phyre2 online software (Fig. 1) showed that scBsAbs connected by Fc, 205C’, or HSA could fold correctly to form complete proteins, and the respective three-dimensional structures of anti-γ-Sm scFv and anti-CD3scFv were not seriously affected. Nevertheless, there were differences in the structures of the separated Fc, 205C’, and HSA peptide fragments. Fc consists of two β-sheets and one α-helix; 205C’ is composed of random coils; and HSA contains two α-helices. The differences in structure among the three linkers may underlie the differences in biological activities of the three resulting scBsAbs.

### Construction and expression of scBsAbs

**Fig. 2:**
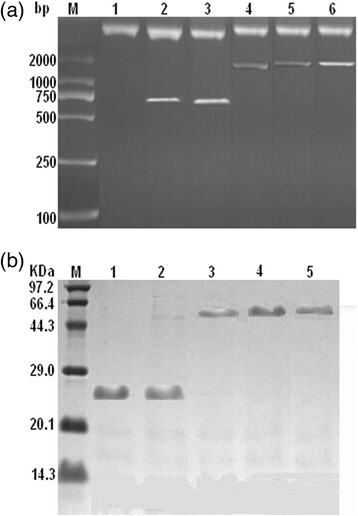
Detection of eukaryotic expression of scBsAbs. **a**. Restriction enzyme cleave identification of pSectag2B-scBsAbs plasmids; **b**. SDS-PAGE analysis of purified scBsAbs

When an expression vector was digested by *Hind*III and *EcoR*I, the bands of γ-Sm scFv and anti-CD3 scFv could be seen as bands of 741 bp and 729 bp, respectively. The length of (Fc)scBsAb, (205C’)scBsAb, and (HSA)scBsAb was ~1.6 kb (Fig. 2a). SDS-PAGE analysis of the purified proteins showed that γ-Sm scFv and anti-CD3 scFv were ~26kD, whereas (Fc)scBsAb, (205C’)scBsAb, and (HSA)scBsAb were ~57kD (Fig. 2b). These results were consistent with the software predictions, indicating that the desired genes were inserted successfully into the expression vector pSectag2B and the corresponding proteins were successfully expressed and purified.

### Antigen binding activity assay

**Fig. 3:**
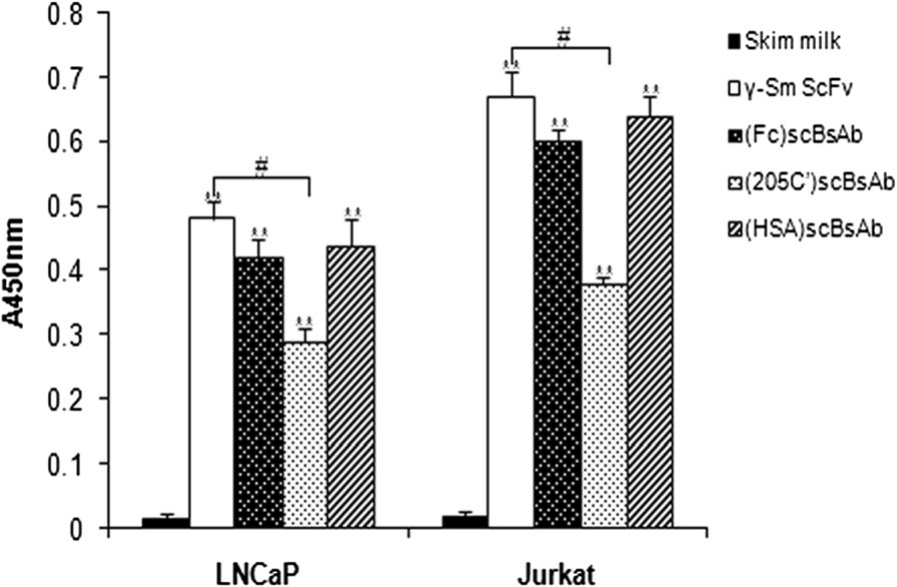
Antigen binding activity of scBsAbs measured by ELISA. Note: vs Skim milk, ^**^ *p* < 0.01; vs γ-Sm ScFv, ^#^ *p* < 0.05

Measurement of the antigen binding activity (Fig. 3) showed that the antigen binding of each scBsAb was much stronger than that of a negative control (skim milk; *P* < 0.01); this result indicated that the three kinds of scBsAb bind to Jurkat cells and to the LNCaP membrane antigen. The antigen binding activity of (205C’)scBsAb was weaker than that of parental scFvs (*P* < 0.05), whereas (Fc)scBsAb and (HSA)scBsAb showed antigen binding similar to that of parental scFvs (*P* > 0.05).

### Pharmacokinetic analysis

A two-compartment model was used for blood clearance curve fitting. The blood pharmacokinetics-fitting equation of each antibody was derived and half-clearance time of the distribution and elimination phase (T_1/2α_ andT_1/2β_) was calculated. Results (Table 1) showed that the T_1/2α_valuesof the three scBsAbs were very similar (and small), indicating that they would spread rapidly within the mouse body. Nevertheless, the differences in T_1/2β_were quite obvious: T_1/2β_ of (HSA)scBsAb was the longest, up to 4.4 h.

**Table 1:** Pharmacokinetic parameters of scBsAb with different interchain linker

scBsAb	Fitted equation	T_1/2α_	T_1/2β_	Goodness of fit
		(min)	(min)	(R^2^)
^125^I -(Fc) scBsAb	y = 8.67e^−0.0041t^ + 4.75e^−0.005t^	16.1	168.7	0.998
^125^I -(205C’) scBsAb	y = 1.86e^−0.0038t^ + 4.75e^−0.0061t^	17.4	119.5	0.995
^125^I -(HSA) scBsAb	y = 14.12e^−0.0432t^ + 2.89e^−0.0035t^	17.2	264.2	0.999

### Therapeutic effects of the scBsAbs

**Fig. 4:**
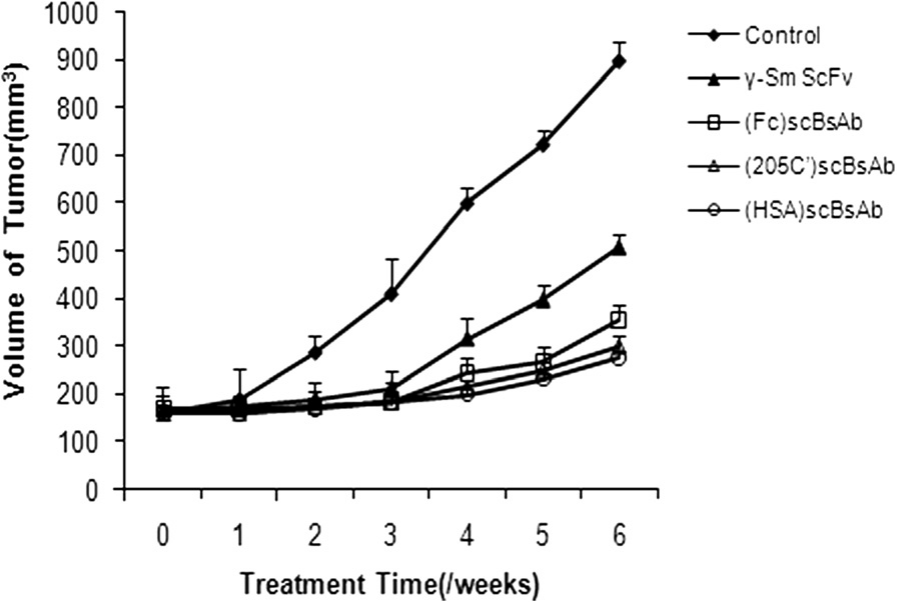
Therapeutic effect analysis of scBsAbs

Five healthy nude mice that received an intravenous injection of each antibody and effector cells showed no obvious pathological changes, indicating that each antibody and each type of effector cells had no obvious adverse effects. Among the 50 nude mice that we used as a prostate cancer model, two died (one died of infection, and the other had an unclear cause of death). The remaining 48 mice were used in the treatment experiment and were subdivided into five groups: the (Fc)scBsAb group (ten mice), (205C’)scBsAb group (ten mice), (HSA)scBsAb group (ten mice), γ-Sm ScFv group (ten mice), and the Control group (eight mice). The results showed that in comparison with the control group, the growth of the prostate tumour in the treatment group was significantly inhibited (*P* < 0.05).In comparison with the γ-Sm ScFv group, the inhibitory effect on tumour growth in groups(Fc)scBsAb, (205C’)scBsAb, and (HSA)scBsAb was much stronger (*P* < 0.05). There were no significant differences among groups (Fc)scBsAb, (205C’)scBsAb, and (HSA)scBsAb (*P* > 0.05; Fig. 4).

## Discussion

Selection of a peptide linker during the design of a scBsAb is an important step. Some studies [15, 16] suggested that the linker should be long and flexible enough to ensure that the two half-molecules connected could have sufficient spatial freedom to perform their functions; on the other hand, the effect on stability of the fusion protein should also be considered. Besides, the size and affinity of an antibody can affect the blood pharmacokinetics. The increase of an antibody’s molecular weight mayslow down its elimination within the body but will weaken tumour penetration [17].

Gustavsson [18] suggested that the length range of linkers for construction of scBsAbs should be 4–44 amino acid residues. Too short a linker may cause spatial steric hindrance within the recombinant molecule, affecting the folding of the protein domains. On the other hand, if the linker is too long, it may enhance undesirable immunogenicity of the antibody. The scFv intrachain linker that we utilised in the present study is a common one: (Gly4Ser)3. Interchain linker Fc is a fragment of human IgG1CH2 (amino acid positions 297–322), 26 amino acids long. HSA is 25 amino acids long and contains no cysteine but many polar amino acids (it comes from the human serum albumin domain D3).205C’ is a 25-amino-acidpeptidefrom a study by Gruber et al. [14]. We analysed the scBsAbs (that were constructed with different peptide linkers) using bioinformatic tools and the conformation was predicted. These data showed that the spatial conformations of the three scBsAbs exactly matched the native conformations of the parental antibodies. The prediction and simulation results may not necessarily reflect the real-life data [19]. Nevertheless, our recombinant BsAbs had a big molecular weight. Close attention should be paid to the type of expression system: prokaryotic or eukaryotic. Prediction of the three-dimensional structure of a protein can form the theoretical foundation for successful expression [20]. Accordingly, we measured the expression and binding activity of the scBsAbs. In addition, the scBsAbs were linked to a His and Myc tag, making the purification and identification of the fusion proteins more convenient. Our results indicated that the three scBsAbs all had a specific binding affinity for Jurkat cells and the LNCaP membrane antigen although the antigen binding activity of (205C’)scBsAb was weaker than that of parental scFvs and the other two scBsAbs. As for pharmacokinetics, the half-clearance time of the elimination phase (T_1/2β_) of (HSA)scBsAb was the longest. Such differences may be due to the differences in the structures of the three interchain linkers.

In fact, multiple factors such as the length of the linker, amino acid composition, glycosylation status, and the suitability of each half-molecule when attached to the linker all affect the function and stability of the fusion protein [21–23]. Kikuchi et al. [24] constructed an anti-CD47 bivalent single-chain MABL sc(Fv)2 with the linker (Gly4Ser)3 of 15 amino acids and showed that the recombinant antibody and parental antibody had almost the same affinity for the antigen and good anti-tumour activity. Goel et al. [25] constructed a bivalent single-chain antibody with good immunological competence with a linker composed of 25 amino acids but didn’t obtain exactly the immunological competence of the parental antibody. According to our results, HSA is the optimal interchain linker and can prolong half-life of the antibody significantly and may substantially improve therapeutic effectiveness of minimolecular antibodies. In a word, the bispecific antibodies make a bridge between the tumor cell and the immune effector cell, which will trigger the cytotoxic responses. The linker is critical for the structure of the whole single-chain bispecific antibody molecule. Application of appropriate linker can supply enough flexibility for the two binding sites of modified antibody to promote the crosslinking between antibody with double antigen sites [26]. The length and composition of linker used to connect the bivalent single-chain antibody often affect the stability and function of the newly formed antibody. The most common linkers contain a combination of glycine and serine residues to provide flexibility and protease resistance [27]. In addition, the middle linker mainly determines the distance between the two antigen-binding sites of the same specificity. Too short distances may preclude bivalent binding of the BsAb to the cell surface, although the probability of the antibody binding to the cell surface bivalently also depends on the antigen density and accessibility. The linker also influences the flexibility of the molecule, the possibility of acquiring the most suitable conformation for binding to the antigen [28].

## Conclusions

According to our study, HSA is the optimal linker for ananti-γ-Sm × anti-CD3 scBsAb; this linker improves the antigen binding activity of such antibodies and prolongs the retention time in comparison with the other linkers: Fc and 205C’. Composition of the interchain linker may affect the function of the scBsAb; therefore, rational selection and design of the linker are crucial for construction of an ideal engineered antibody.

## Materials and methods

### Materials

The pUC18 and pSectag2B plasmids, restriction endonucleases, and DNA ligase were all purchased from TaKaRa Bio Inc. (Tokyo, Japan); plasmids pUC18-γ-Sm ScFv and pUC18-CD3 ScFv were constructed and stored in our laboratory. JM109 competent cells and HeLa cells were purchased from Tiangen Biotech Co., Ltd. (Beijing, China). The Lipofectamine2000 transfection kit was from Invitrogen Corporation (CA, USA). The ELISA kit was purchased from Shanghai Ding Biological Technology Co., Ltd. (Shanghai, China). Balb/c mice and nude mice were provided by the Animal Experimental Center of Zhengzhou University.

### Design and synthesis of scBsAbs

The scBsAbs was designed on the basis of nucleotide sequences of anti-γ-Sm scvF [29] and anti-CD3 scFv [30]. The intrachain peptide linkers of scFv consisted of the common linker: (Gly4Ser)_3._ The interchain peptide linker between the two kinds of scFvs was Fc, 205C’, or HSA. Restriction site *Hind*III and site *EcoR*I were introduced at one of the ends of the complete DNA sequence of the scBsAb. The designed gene sequences were synthesised by Shanghai Sangon Biotech Co., Ltd.

### Prediction of spatial conformation

The Phyre 2 online software was used to construct three-dimensional models of (Fc)scBsAb, (205C’)scBsAb, and (HSA)scBsAb. We analysed interchain linker sequences when combined with ten amino acid residues close to the opposite ends of the sequence. Differences in spatial conformation of scBsAbs were compared.

### Construction of a eukaryotic expression vector

The synthesised scBsAb gene fragments were cloned into the pUC18 plasmid for screening of successful positive clones. By sequencing, we found that the scBsAb gene fragments had been inserted into the pUC18 vector successfully, and the recombinant sequences pUC18-(Fc)scBsAb, pUC18-(205C’)scBsAb, and pUC18-(HSA)scBsAb were successfully assembled. Enzymes *Hind*III and *EcoR*I were used to digest pUC18-(Fc/205C’/HSA)scBsAb, pUC18-γ-Sm ScFv, and pUC18-CD3 ScFv. The purified scBsAb(Fc/205C’/HSA) and scFv gene fragments were linked by means of T4 ligase with pSectag2B (secretory eukaryotic expression vector with a 6 × His and Myc tag at downstream polyclonal loci) digested by *Hind*III and *EcoR*I, which were introduced into JM109 competent cells. A positive clone was identified after screening and named pSectag2B-(Fc)scBsAb, pSectag2B-(205C’)scBsAb, pSectag2B-(HSA) scBsAb, pSectag2B-γ-Sm ScFv, or pSectag2B-CD3 ScFv. Then the plasmids were isolated and prepared in sufficient quantities.

### Cultivation of HeLa cells and plasmid transfection

Cryovials of HeLa cells were taken out of storage quickly and thawed in a shaking bath at 37 °C. Under aseptic conditions, a suspension of HeLa cells was transferred to a centrifuge tube. A 10-fold volume of the RPMI1640 culture medium was added, and the mixture was centrifugated at 350 g for 10 min. The cells were resuspended in the complete medium gently and then seeded in a 25-cm^2^ culture flask. After cultivation at 37 °C and 5 %CO_2_ with saturated humidity for 24 h, the medium was refreshed for further cultivation. When the HeLa cells attained 80 % confluence, they were subcultured1:3. On the day before transfection, the cells were harvested by trypsinisation. The cell concentration was adjusted, and the cells were seeded in a 12-well plate containing the culture medium without antibiotics at the density 2.5 × 10^5^ cells/well. We diluted 1.5 μL of Lipofectamine2000 to 20 μL with DMEM and then mixed it with 20 μL of a pSectag2B-scBsAb or pSectag2B-ScFv solution (1 μg/μL); the mixture was incubated for 5 min and then transfected into HeLa cells. Meanwhile, the pSectag2B plasmid without the antibody gene was transfected into a separate batch of HeLa cells, which served as a control. The medium was changed after 5-h transfection. After cultivation of the cells for 72 h in a CO_2_ incubator at 37 °C, the culture medium was collected for isolation of the antibodies. The proteins were purified by Ni^2+^-NTA agarose, and 25-μL aliquots were used for SDS-PAGE analysis.

### Measurement of antigen binding activity of the scBsAbs by ELISA

Jurkat cells or the LNCaP membrane antigen at 50 μg/mL were used to coat an ELISA plate (incubation at 4 °C overnight). 1 % solution of BSA was used to block nonspecific antigens at 37 °C for 2 h, and then 100 μL of a solution of various antibodies (20 μg/mL) was added, with subsequent incubation at 37 °C for 2 h. After washes with TPBS and PBS (three times each), 100 μL of a mouse anti-human Myc antibody (1:2,000) was added, and the mixture was incubated at 37 °C for 1 h. After three washes each with TPBS and PBS, 100 μL of a horseradish peroxidase-conjugated goat anti-mouse IgG antibody (1:5,000) was added and incubated at 37 °C for 1 h, followed by three washes each with TPBS and PBS. Finally, 100 μL of the tetramethylbenzidine (TMB) substrate (1 mg/mL TMB, 0.1 mM sodium acetate pH6.0, and 0.006 %H_2_O_2_) was used for visualisation (incubation for 20 min). We added 50 μL of 1Msulphuric acid to each well to terminate the reaction. Absorbance at 450 nm was measured, with reference wavelength 630 nm.

### An assay of blood pharmacokinetics

The iodogen method was used for radioiodination of the antibodies. Thirty Balb/c mice were randomly distributed into three groups. The mice in each group received an injection of 0.5 mL of^125^I-scBsAb through the caudal vein. Then, 10 μL of blood was collected from the tail vein at the time points0, 5 min, 15 min, 30 min, and 24 h after the injection. After that, radioactivity counts were recorded, and blood clearance parameters were calculated.

### Preparation of effector cells and treatment with scBsAbs

Density gradient centrifugation was used to isolate peripheral blood mononuclear cells (PBMCs) from normal blood after heparinisation. PBMCs were precooled and resuspended in PBS, then centrifuged at 350 g for 10 min. With the supernatant discarded, the RPMI1640 culture medium containing 10 % foetal calf serum, 2 mM glutamine, 100U/mL penicillin, and 100 μg/mL streptomycin was added to resuspend the cells, and the cell suspension was then transferred to 250-mL cell culture flasks, placed in an incubator with 5 % CO_2_ at 37 °C for 1-h incubation. Non-adherent cells were transferred into a new culture flask, with the culture medium changed and 150U/mL IL-2 added. After incubation at 37 °C for 3d, the culture medium was changed again and the concentration of IL-2 was adjusted to 10^5^U/mL. Later, the culture medium was changed every three days, and the effector cells were obtained after constant cultivation for eight weeks.

First, five healthy nude mice received an injection (200 μg) of one of the antibodies (each mouse per antibody) and 10^7^ effector cells, for analysis of adverse effects of each kind of antibody and effector cells. Fifty nude mice were subdivided into five groups randomly (10 per group): the (Fc)scBsAb group, (205C’)scBsAb group, (HSA)scBsAb group, γ-Sm ScFv group, and the Control group. Nude mice in each group received ventral subcutaneous injection of 10^7^ LNCaP cells. When gross tumour volume was ~100 mm^3^, the treatment plan for each group started along with monitoring. Mice in the Control group received only the intravenous injection of 10^7^effector cells, and those in the treatment group received an intravenous injection of 10^7^effector cells and 200 μg of one of the antibodies. The volume of the tumour was measured once a week. The formula for calculation of the tumour volume was length × width^2^ × 0.52. After six weeks, the nude mice were killed by CO_2_ asphyxiation.
